# MALDI-TOF Mass Spectrometry Detection of Pathogens in Vectors: The *Borrelia crocidurae*/*Ornithodoros sonrai* Paradigm

**DOI:** 10.1371/journal.pntd.0002984

**Published:** 2014-07-24

**Authors:** Aurélien Fotso Fotso, Oleg Mediannikov, Georges Diatta, Lionel Almeras, Christophe Flaudrops, Philippe Parola, Michel Drancourt

**Affiliations:** 1 URMITE, UMR 6236, CNRS 7278, IRD 198, INSERM 1095, Méditerranée Infection, Faculté de Médecine, Aix-Marseille Université, Marseille, France; 2 URMITE, UMR, IRD 198, Campus IRD Ham Manisty, Dakar, Senegal; 3 Pôle de Maladies Infectieuses, Assistance Publique-Hôpitaux de Marseille, CHU Timone, Marseille, France; University of California at Davis, United States of America

## Abstract

**Background:**

In Africa, relapsing fever borreliae are neglected vector-borne pathogens that cause mild to deadly septicemia and miscarriage. Screening vectors for the presence of borreliae currently requires technically demanding, time- and resource-consuming molecular methods. Matrix-assisted laser desorption/ionization time-of-flight mass spectrometry (MALDI-TOF-MS) has recently emerged as a tool for the rapid identification of vectors and the identification of cultured borreliae. We investigated whether MALDI-TOF-MS could detect relapsing fever borreliae directly in ticks.

**Methodology/Principal Findings:**

As a first step, a *Borrelia* MALDI-TOF-MS database was created to house the newly determined Mean Spectrum Projections for four Lyme disease group and ten relapsing fever group reference borreliae. MALDI-TOF-MS yielded a unique protein profile for each of the 14 tested *Borrelia* species, with 100% reproducibility over 12 repeats. In a second proof-of-concept step, the *Borrelia* database and a custom software program that subtracts the uninfected *O. sonrai* profile were used to detect *Borrelia crocidurae* in 20 *Ornithodoros sonrai* ticks, including eight ticks that tested positive for *B. crocidurae* by PCR-sequencing. A *B. crocidurae*-specific pattern consisting of 3405, 5071, 5898, 7041, 8580 and 9757-m/z peaks was found in all *B. crocidurae*-infected ticks and not found in any of the un-infected ticks. In a final blind validation step, MALDI-TOF-MS exhibited 88.9% sensitivity and 93.75% specificity for the detection of *B. crocidurae* in 50 *O. sonrai* ticks, including 18 that tested positive for *B. crocidurae* by PCR-sequencing. MALDI-TOF-MS took 45 minutes to be completed.

**Conclusions/Significance:**

After the development of an appropriate database, MALDI-TOF-MS can be used to identify tick species and the presence of relapsing fever borreliae in a single assay. This work paves the way for the use of MALDI-TOF-MS for the dual identification of vectors and vectorized pathogens.

## Author Summary

In Africa, relapsing fever borreliae are neglected vector-borne infections that cause mild to deadly septicemia and miscarriage. The causative relapsing fever borreliae are transmitted by the bite of soft ticks, except for *Borrelia recurrentis* which is transmitted by body lice. Screening vectors for these relapsing fever borreliae currently relies on time- and resource-consuming methods such as polymerase chain reaction-based method. Here, we applied matrix-assisted laser desorption/ionization time-of-flight mass spectrometry (MALDI-TOF-MS) to the rapid detection of borreliae in ticks. As a first step, we created a *Borrelia* MALDI-TOF-MS database and we detected *B. crocidurae* in *Ornithodoros sonrai* ticks. As a blind validation step, the 45-minute MALDI-TOF-MS exhibited a 88.9% sensitivity and a 93.75% specificity for the detection of *B. crocidurae* in 50 *O. sonrai* ticks including 18 ticks detected positive for *B. crocidurae* by PCR-sequencing. These findings provide the proof-of-concept that MALDI-TOF-MS can be used to identify tick species and the presence of relapsing fever borreliae. This technique could be translated for field applications.

## Introduction

The genus *Borrelia* is composed of bacterial pathogens responsible for relapsing fever and Lyme borreliosis [1]. Whereas the Lyme disease agents *Borrelia burgdorferi* [2], *Borrelia garinii* [3], *Borrelia afzelii* [4] and *Borrelia valaisiana* [5] are transmitted by hard ticks, the relapsing fever borreliae are transmitted by soft ticks [6], [7] and lice (in the case of *Borrelia recurrentis*) [8].

In Africa, four cultured species, *Borrelia crocidurae*, *Borrelia duttonii*, *Borrelia recurrentis* and *Borrelia hispanica*, and several not-yet cultured species are circulating in vector populations [1]. Screening vectors for these relapsing fever borreliae currently requires the use of technically demanding, time- and resource-consuming molecular methods [9], [10]. However, matrix-assistedlaser desorption/ionization time-of-flight mass spectrometry (MALDI-TOF-MS) has emerged as a new, easy technique that can hasten the identification of bacteria and archaea [11], [12]. In particular, MALDI-TOF-MS has been used to identify spirochetes of the genus *Leptospira* [13] and *Brachyspira* [14], and recently cultured *Borrelia* spp. [15]. Also, MALDI-TOF-MS has recently been used to identify vectors [16]–[18].

In this study, we investigated whether MALDI-TOF-MS could further detect relapsing fever borreliae directly in their tick vectors. More specifically, we developed a step-by-step approach, first creating a *Borrelia* MALDI-TOF-MS database, then performing a proof-of-concept *B. crocidurae* detection in *Ornithodoros sonrai* ticks, and finally, blindly testing the MALDI-TOF-MS approach to detect *B. crocidurae* in *O. sonrai* ticks.

## Materials and Methods

### Borreliae and ticks

Fourteen *Borrelia* spp. were grown at 32°C in Barbour-Stoenner-Kelly-H (BSK-H) medium (Sigma, Saint Quentin Fallavier, France) supplemented with 10% heat-inactivated rabbit serum (Eurobio, Courtaboeuf, France) (Table 1). Dark-field microscopic observation was performed to ensure the absence of any contaminants and to verify the richness and viability of the culture. The identification of the growing borreliae was performed by PCR-sequencing the flagellin gene, as previously reported [19]. The *Borrelia* culture was centrifuged at 13,000 g for 10 min at room temperature, and the pellet was washed twice with 1 mL high performance liquid chromatography grade water (VWR International, Fontenay-sous-Bois, France) and then suspended at 10^4^ spirochetes/mL in this water before MALDI-TOF-MS analysis.

**Table 1:** *Borrelia* species used to establish a MALDI-TOF-MS reference database.

Species	Strain	MALDI-TOF-MS identification	Score Value
*B. burgdorferi*	B31	*B. burgdorferi*	2.34
*B. recurrentis*	A1	*B. recurrentis*	2.39
*B. crocidurae*	Achema	*B. crocidurae*	2.45
*B. duttonii*	Ly	*B. duttonii*	2.60
*B. lusitaniae*	Poti B2	*B. lusitaniae*	2.67
*B. japonica*	HO14	*B. japonica*	2.65
*B. afzelii*	PKo	*B. afzelii*	2.27
*Borrelia sp.*	CA 28 (“genomospecies 2”)	*B. genomosp.*	2.36
*B. andersonii*	19952	*B. andersonii*	2.50
*B. garinii*	PBi	*B. garinii*	2.56
*B. californiensis*	CA446	*B. californiensis*	2.34
*B. valaisiana*	VS116	*B. valaisiana*	2.25
*B. hermsii*	BH0147	*B. hermsii*	2.49
*B. turcica*	IST7	*B. turcica*	2.15

Fifty ticks were collected in Senegal. Total DNA was extracted from the body of the tick by using the EZ1 DNA Tissue kit and the EZ1 apparatus (Qiagen, Courtaboeuf, France) for further PCR-sequencing-based investigations. Ticks were identified as *O. sonrai* (a species not registered as an endangered species) by 16S rRNA gene sequencing, as previously described [20]. The ticks were tested for the presence of *B. crocidurae* by *glpQ* gene real-time PCR using a Ct≤35 cut-off [21], and 18/50 (36%) ticks were found to be infected.

### Borreliae MALDI-TOF-MS database

A 1 µL aliquot of the suspension was deposited onto a spot on an MSP 96 target polished steel micro Scout target plate (Bruker Daltonics, Wissembourg, France). After air-drying, 1.5 µL of matrix solution (a saturated solution of alpha-cyanohydroxycinnaminic acid in 50% aqueous acetonitrile containing 2.5% trifluoroacetic acid) was added, and the plate was air-dried for 15 min before being processed in the mass spectrometer. A total of 12 spots were deposited for each *Borrelia* strain, and this manipulation was repeated in two independent runs. On each plate, *Escherichia coli* DH5 alpha (Bruker Daltonics) was used as a positive control, and non-inoculated BSK-H medium and non-inoculated-matrix solution were used as negative controls. The analysis was conducted using a Microflex LT spectrometer (Bruker Daltonics), and the spectra were recorded in a linear, positive ion mode with an acceleration voltage of 20 kV. The spectra were collected as a sum of 240 shots across a spot. The preprocessing and identification steps were performed using the manufacturer's parameters. For each of the 14 *Borrelia* species, a consensus pattern referred to as the Mean Spectrum Projection (MSP) was obtained by the Biotyper MSP Creation Standard Method (Bruker Daltonics). To assess the reproducibility of the MALDI-TOF-MS profiling, the 14 *Borrelia* strains included in the database were further blindly analyzed by MALDI-TOF-MS as described above. For each strain, 12 spots were analyzed, and the 12 spectra obtained were compared with the *Borrelia* MSP database. The results of the pattern-matching process were expressed as an identifying score varying from 0 to 3. A score of between 3 and 1.9 indicated a species level identification; a score of 1.9–1.7 indicated a genus level identification; and a score of <1.7 was regarded as an unreliable identification [22]. The MSP spectra from Borrelia spp. were used to generate a dendogram with the MALDI Biotyper 2.0 software (Bruker Daltonics).

### MALDI-TOF-MS detection of *B. crocidurae* in ticks

A total of 20 *O. sonrai* ticks, including 8 *B. crocidurae*-infected ticks, were studied. Each tick was placed in a 1.5 mL microcentrifuge tube, frozen at −20°C for 30 min [18], [23], rinsed once with distilled water and dried with paper. Four legs were removed with scalpels and manually homogenized in 60 µL of 70% formic acid and 60 µL of 50% acetonitrile in 1.5 mL microcentrifuge tubes using pellet pestles (Thermo Fischer Scientific, Courtaboeuf, France). All of the homogenates were centrifuged at 13,000 g for 20 s, and 1 µL of the supernatant was spotted onto a steel target plate in quadruplicate. Using in-house subtraction software, the MSP pattern of non-infected *O. sonrai* was removed from the pattern of infected ticks. The software normalizes the spectra comparing common peaks in infected and uninfected ticks and then generates the MSP spectra before performed the subtraction. After subtraction, the list of remaining differential masses (m/z) was compared with the *B. crocidurae* MSP.

### Blind MALDI-TOF-MS detection of *B. crocidurae* in *O. sonrai* ticks

The 50 *O. sonrai* ticks were coded and blindly tested for the presence of *B. crocidurae* using the *Borrelia* database and the subtraction software described above. After blind MALDI-TOF-MS analysis, the codes were unmasked to compare the MALDI-TOF-MS assay results with the PCR-sequencing-based results. Any surprising result was double-checked by performing MALDI-TOF-MS and PCR-sequencing detection again.

## Results

### MALDI-TOF-MS *Borrelia* database

Negative controls yielded no identifiable patterns while the positive controls yielded *E. coli* with identification scores from 1.9–2.3. Each of the 14 *Borrelia* reference isolates yielded a unique reproducible protein profile combining 25 to 123 peaks (Table S1). Each profile differed from the ones in the Bruker Daltonics database (V3.1.2.0) and each profile was specific for each species of *Borrelia* (Table S1). Further blind test correctly identified all of the 14 *Borrelia* strains, with identification scores from 2.15–2.67 after incorporation of the reference spectra (Table 1).

### MALDI-TOF-MS detection of *B. crocidurae* in *O. sonrai* ticks

**Figure 1:**
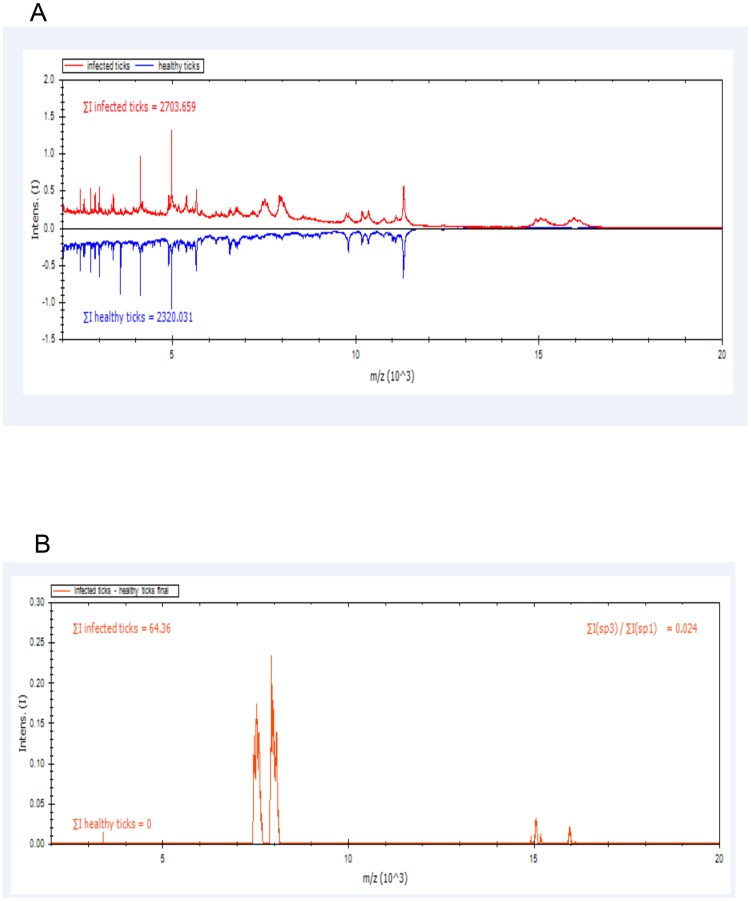
Subtraction of infected-tick pattern minus non-infected-tick pattern by our subtraction software (1A). Residual spectrum obtained after the subtraction of the non-infected tick pattern from the infected tick pattern (1B).

**Figure 2:**
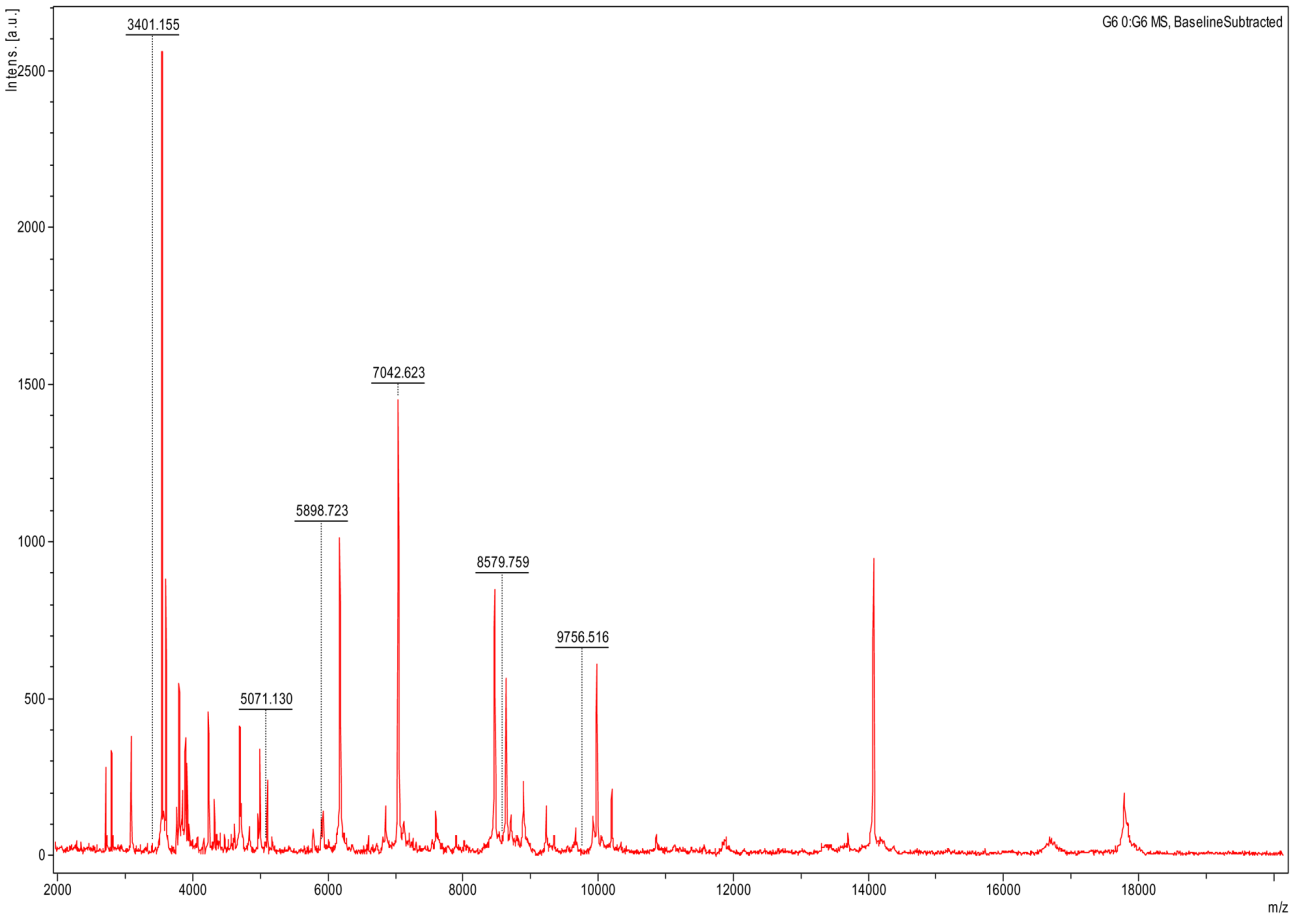
A 6-peak pattern for the specific detection of *Borrelia crocidurae* in *O. sonrai* ticks.

The 12 un-infected *O. sonrai* ticks yielded no matches, as the database we used did not contain a reference spectrum for this soft tick species; we therefore added this new reference MSP in the ticks' database being constructed in our laboratory [17]. The eight infected *O. sonrai* ticks consistently yielded a specific pattern of six peaks of 3405, 5071, 5898, 7041, 8580 and 9757 m/z. After subtraction of the *O. sonrai* MSP, none of the 12 un-infected ticks yielded such a pattern. This 6-peak pattern was found in the *B. crocidurae* MALDI-TOF-MS pattern (Figures 1, 2). Using this 6-peak pattern, *B. crocidurae* was detected in 100% of infected ticks and 0% of non-infected ticks.

### Blind detection of *B. crocidurae* in *O. sonrai*

During the blind test, 100% of the 50 tested ticks were identified as *O. sonrai* (after the corresponding MSP had been added to the database). Furthermore, the 6-peak *B. crocidurae* pattern was blindly detected in 16/18 (sensitivity, 88.9%) infected ticks and in 2/32 (6.25%) non-infected ticks (specificity of 93.75%) (Table 2). Re-testing these four ticks with both *glpQ*–qPCR and MALDI-TOF-MS yielded the same results as in previous experiment.

**Table 2:** MALDI-TOF-MS detection of *B. crocidurae* in 50 *O. sonrai* ticks collected in Senegal.

		MALDI-TOF-MS	
		+	−
glpQ qPCR	18 +	16	2
	32 −	2	30

“+” denotes a positive result; “−” denotes a negative result.

## Discussion

For ten years, MALDI-TOF-MS has revolutionized the routine identification of bacteria [11], [22], [24] and archaea [12], but few studies have examined its application to spirochetes, including *Leptospira* spp. [13] *Brachyspira* spp. [14] and *Borreliae* spp. [15]. Here, using a simple protocol consisting of only one centrifugation step, 14 *Borrelia* isolates yielded an interpretable, identifying protein pattern. Identification was achieved starting from only 10^4^ *Borrelia*/mL, in the same range as the 10^5^ *Leptospira*/mL previously reported [13]. These data completely confirm a recently published study showing that cultured *Borreliae* are identifiable by MALDI-TOF-MS [15]. The MSP for the 14 Borrelia isolates described herein have been deposited into our freely available Mediterranée Infection Institute website database (http://www.mediterranee-infection.com/article.php?laref=256&titre=urmsdatabase) to assist other scientists with their identification work. As the reliability of MALDI-TOF-MS bacterial identifications depends on the quality of the database [22], we carefully validated the reproducibility of the *Borrelia* spectra. No misidentifications were discovered when all of the *Borrelia* strains analyzed in this study were matched with non-borreliae spirochetes (*Leptospira* and *Brachyspira*). Further enrichments of the database and the creation of additional super spectra using several genetically/morphologically different strains of the same species will likely increase the power of MALDI-TOF-MS for the identification of borreliae. We also added an MSP for *O. sonrai* to the tick MALDI-TOF-MS database that we are building [17].

Using this extended database and a piece of subtraction software, MALDI-TOF-MS allowed for the blindly identification of *O. sonrai* ticks and detection of *B. crocidurae* in these ticks within 45 minutes. Interestingly, infection by *B. crocidurae* did not hamper the MALDI-TOF-MS identification of the infected *O. sonrai*. Moreover, the 6-peak signature found in *B. crocidurae* infected ticks is comprised of six proteins that are specifically found in *B. crocidurae*. Therefore, this 6-peak signature is not an unspecific pattern due to any ticks' infection or inflammatory response to *Borrelia*, but indeed a signature specific to *B. crocidurae*. Using a 96-spot plate, a total of 23 ticks could be screened in the same experiment, including controls and quadruplicate spots per tick. These data indicate that MALDI-TOF-MS can be used for the rapid, one-shot identification of ticks and tick-borne borreliae. This is the first report of dual identification of vector and vectorized pathogen at once, although a few vectors, including mosquitoes and ticks, have been previously identified by MALDI-TOF-MS [16]–[18].

Further studies are warranted to extend this concept to other pathogens and other vectors. Automation of the differential peak list interpretation would help hasten the field implementation of MALDI-TOF-MS as a first-line tool for the rapid identification of vectors and vectorized pathogens.

## Supporting Information

Table S1 List of peaks (m/z) detected by MALDI-TOF-MS for each of the 14 *Borrelia* species under study.
(XLSX)
